# Chemoembolization of Hepatocellular Carcinoma with Hepasphere 30–60 μm. Safety and Efficacy Study

**DOI:** 10.1007/s00270-013-0777-x

**Published:** 2013-11-22

**Authors:** Katerina Malagari, Maria Pomoni, Hippokratis Moschouris, Alexios Kelekis, Angelos Charokopakis, Evanthia Bouma, Themistoklis Spyridopoulos, Achilles Chatziioannou, Vlasios Sotirchos, Theodoros Karampelas, Constantin Tamvakopoulos, Dimitrios Filippiadis, Enangelos Karagiannis, Athanasios Marinis, John Koskinas, Dimitrios A. Kelekis

**Affiliations:** 1 2nd Department of Radiology, University of Athens, Athens, Greece; 2 Imaging and Research Unit, University of Athens, Athens, Greece; 3 Department of Radiology, Tzanion Hospital, Athens, Greece; 4 Research Academy of University of Athens, Athens, Greece; 5 1st Department of Surgery, Tzanion Hospital, Piraeus, Greece; 6 2nd Department of Internal Medicine and Hepatology, University of Athens, Athens, Greece; 7 Imaging and Research Unit, 2nd Department of Radiology, University of Athens, Athens, Greece

**Keywords:** Conventional chemoembolization (c-TACE), Hepatocellular carcinoma (HCC), HepaSphere, Drug eluting chemoembolization

## Abstract

**Background:**

This study examined the safety, pharmacokinetics, and efficacy of transarterial chemoembolization of hepatocellular carcinoma (HCC) using a newly developed size of a superabsorbent polymer drug-eluting embolic material.

**Methods:**

Forty-five patients with documented HCC (Child–Pugh score A/B: 55.5 %/44.5 %) were embolized with HepaSphere microspheres 30–60 μm with escalation of lesion, dose, and frequency of re-embolization. Local response was evaluated with modified response evaluation criteria in solid tumors (mRECIST). Plasma levels of doxorubicin were measured in 24 patients at baseline and at 5, 20, 40, 60, and 120 min, at 6, 24, and 48 h, and at 7 days, respectively, to determine doxorubicin in plasma (Cmax) and area under the curve (AUC). Measurements of three patients who underwent lipiodol-based conventional chemoembolization (c-TACE) were also performed.

**Results:**

TACE with HepaSphere was well tolerated with an acceptable safety profile and no 30-day mortality. Response rates were calculated on intention-to-treat basis with complete response (CR) in 17.8 % reaching 22.2 % for the target lesion. Overall partial response (PR) was seen in 51.1 %, stable disease in 20 %, and progressive disease in 11.1 % of patients. Overall objective response (CR + PR), including patients treated at all dosages of doxorubicin, was seen in 68.9 % of cases. After a median follow-up of 15.6 months, 1-year survival is 100 %. Doxorubicin AUC was significantly lower in patients with HepaSphere 30–60 μm (35,195 ± 27,873 ng × min/ml) than in patients with conventional TACE (103,960 ± 16,652 ng × min/ml; *p* = 0.009). Cmax was also significantly lower with HepaSphere 30–60 μm (83.9 ± 32.1 ng/ml) compared with c-TACE (761.3 ± 58.8 ng/ml; *p* = 0.002).

**Conclusion:**

HepaSphere 30–60 μm is an effective drug-eluting embolic material with a favourable pharmacokinetic profile.

## Introduction

Level 1 evidence has proved that transcatheter chemoembolization (TACE) has a positive impact on survival of patients with hepatocellular carcinoma (HCC) [1, 2]. During the last 8 years, drug-eluting embolic agents have been tested as new devices with encouraging results on local response [3–7], and level 1 evidence on decreased toxicity compared with lipiodol-based conventional TACE (c-TACE) has been provided [8]. In addition, single series have shown increased rates of long-term survival [9, 10]. In the majority of the studies on drug-eluting embolic material sizes >300 μm were used [3–5, 8, 11], while 100–300-μm diameters were used in a few centers showing trends of superiority of smaller diameters (100–300 μm) compared with larger sizes [6, 7, 9, 12]. In addition, in a large series of patients a recent study comparing safety of various sizes of DC Bead (Biocompatibles, BTG) found that smaller diameters (100–300 μm) were not associated with increased complications [13]. The smaller calibers seem to be attractive because they cause more distal embolization within the tumor where they deliver the chemotherapeutic locally [9, 12, 13].

HepaSphere 30–60 μm is a new size of a loadable microsphere that has a dry caliber of 30–60 μm that expands to 166–242 (197 ± 31) μm in saline and 145–213 (148 ± 45) μm after loading with doxorubicin [14]. In this study, the clinical application of HepaSphere 30–60 μm (Biosphere, Merit) in the treatment of HCC not amenable to curative treatments was examined with primary end points, including safety and local response, after three embolization sessions. Furthermore, a pharmacokinetic study of the levels of doxorubicin in plasma compared with c-TACE has been performed.

## Materials and Methods

This is a prospective study that was launched in June 2011 after receiving Institutional Review Board approval and was completed in February 2013.

### Patient Selection, Schedule of Treatments, and Escalation

#### Eligibility

Patients with documented HCC according the American Association for the Study of Liver Diseases (AASLD) guidelines were included [15, 16]. Patient population and stratification in this study followed the Barcelona Clinic Liver Cancer (BCLC) staging system as recommended in the AASLD-Journal of the National Cancer Institute guidelines for trial design in HCC [17]. Patients enrolled were BCLC B stage HCC and patients with BCLC A not amenable to curative treatments [radiofrequency ablation (RFA) or surgery] due to systemic or anatomic reasons. Liver function prerequisites for enrollment included bilirubin <3 mg/dl and aspartate amino transferase (AST) and alanine amino transferase (ALT) levels <270 IU/ml. Previous treatments with local ablation or resection were not criteria for exclusion. Nontreatable arteriovenous shunts, extrahepatic disease, portal thrombosis (main trunk), and patients receiving angiogenesis agents were excluded.

#### Escalation

The first six patients had lesions ≤6 cm (sum of diameters) with a targeted dose of 50 mg of doxorubicin and repeat embolization performed in 7–8 weeks; patients no. 7–10 had the same sum of diameter of targets and intended drug dosage but were embolized 4–5 weeks after the previous embolization if complete response (CR) was not achieved. Patients no. 11–15 included lesions >6 cm (sum of diameters), intended doxorubicin dosage of 100 mg, and repeat embolization performed in 7–8 weeks; patients no. 16–25 had the same diameter of lesions and intended drug dosage but were embolized 4–5 weeks after the previous embolization if CR was not achieved. The remaining patients no. 26–45, included those with any tumor diameter, an intended dose of 100 mg of doxorubicin, and repeat embolization performed 4–5 weeks after the previous session. Patients in this study were scheduled for three embolization sessions unless CR was achieved with fewer sessions; after these scheduled sessions, they were treated with embolization only on demand.

### Imaging

Baseline imaging and imaging 4 weeks after each procedure was performed with multi-detector computed tomography (MDCT) or magnetic resonance imaging (MRI) and contrast-enhanced ultrasound (CEUS). CEUS was never performed as a sole imaging study but rather as a complimentary study to MDCT or MRI.

Image acquisition was performed according to the guidelines proposed by Lencioni and Llovet [17] with MDCT or MRI including arterial phase and portal venous phase and delayed imaging in the equilibrium phase. MDCT was performed with thin contiguous slices of 5-mm thickness and 5-mm reconstruction interval with a 64-slice multidetector scanner (Brilliance-64; Philips Medical Systems) at three-phases (arterial, portal and equilibrium). MRI was performed with a 3T magnet (Achiva Tx Multi Transmit; Philips Medical Systems) and included T1 fat-suppressed images, T2 fat-suppressed sequences, and dynamic fat-suppressed T1 sequences with gadolinium enhancement and diffusion-weighted axial images. CEUS was performed with a Siemens Acuson, Sequoia 512 equipment, using Sonovue (Bracco) as an enhancing agent with a compatible logistic program.

### Response Assessment

Local response assessment was performed with the modified response evaluation criteria in solid tumors framework criteria (mRECIST) criteria [17, 18]. Image evaluation was performed by K. M. and M. P. and included overall response assessment, target lesion response, nontarget lesion response, and new lesions as required by mRECIST criteria [18]. Only well-delineated, arterially enhancing lesions <1 cm in diameter were selected as target lesions as suggested by mRECIST criteria. This greatly facilitated results assessment. Nontarget lesions were those that were poorly demarcated or exhibited atypical enhancement. In this category (nontarget), the disappearance of any intratumoral enhancement was considered as CR, and persistence of enhancement was classified as incomplete response or stable disease (SD) by mRECIST criteria. It was difficult for the readers in some cases to distinguish between these two categories (incomplete or stable); therefore, if this was the case, it was classified as nonprogressive disease. New lesions included only new foci of at least 1 cm as suggested by mRECIST criteria. All cases with new lesions were classified as progressive disease (PD) if MDCT or MRI and CEUS showed a typical hypervascular pattern. The overall assessment was the result of combined response of target and nontarget lesions as well as new lesions. Regarding nonmeasurable lesions, the response was limited to either CR or non-PD due to difficulties in discrimination between incomplete response and SD. The local response was measured at 1 month after the last embolization (after the completion of two or three embolization sessions; two when CR had already been achieved). Response assessment was completed with alpha fetoprotein (AFP) levels at 1 month after embolization, and these values were compared with baseline AFP.

### Safety

Safety was monitored with blood analysis as well as the evaluation of bilirubin, liver enzymes, creatinine levels, and serum amylase at baseline and at the time of imaging (4 weeks after each embolization session). In addition, on imaging, nontarget embolization and other potential complications were recorded, including ascites, pleural fluid, gallbladder wall thickening, and presence of bilomas or abscesses. Additional unscheduled visits were recorded as was the need for additional medication. Patency of feeding vessels on repeat embolization was also recorded. Toxicity and adverse events were recorded and classified according to the National Cancer Institute Common Terminology Criteria for Adverse Events (version 3.0) [19, 20].

### Technique of Embolization

#### Catheterization

Catheterization of the Haller tripod and superior mesenteric artery was performed with a Simmons 5F or Cobra 5F catheter for vascular mapping followed by catheterization of the feeding vessels to the tumor selectively or superselectively individually using a microcatheter [Progreat 2.7 or 2.4 (Terumo); Renegate HiFlo/Fathom (Boston Scientific); or Microferret 3F or Cantata 2.5F (Cook)]. In difficult cases/small lesions or relatively hypovascular tumors with faint blush intraprocedural, CEUS was performed to document the correct position of the microcatheter and inclusion of the target in the area of injection using a technique described previously [21]. Special attention was given to angiographic controls that were made after a few minutes from the injection of the embolic agent to avoid reflux of the microspheres. The end point of embolization was near stasis; after it was achieved, a waiting time of 3–5 min followed for the microspheres to be redistributed in the feeding vessels; and then more embolic material was injected until back-flow was seen fluoroscopically.

#### Preparation of HepaSphere Microspheres 30–60 μm

Each vial of HepaSphere 30–60 μm was loaded with 25 mg of doxorubicin diluted in 20 ml of normal saline for the first 10 cases and 50 mg of doxorubicin per vial for patients no. 11–45 (each vial was equally loaded). Loading was performed in 2 steps as suggested by the manufacturer: first 10 ml of the solution of doxorubicin was added to the HepaSphere vial and agitated frequently for 10 min, and then the remaining 10 ml were added. The vial was agitated periodically for 1 h to complete the ionic bonding of the doxorubicin. After the loading period, all supernatant was extracted, and an equal quantity of nonionic contrast diluted with saline (50:50) was added to form the final suspension for injection. Overall, the final injectable volume for each vial was 30 ml. Before beginning the injection of the suspension, 100 μm of glyceryl trinitrate (nitrolingual) were injected through the microcatheter at the target vessel to achieve the maximum vessel dilatation, and the microcatheter was flushed with hyperheparinized saline (2.500 IU/500 ml of flushing saline). Slow incremental injection of the suspension followed at a rate of 1–3 ml/min until obliteration of the neovascularity. When initial stasis had been achieved, there was a further wait for 3–5 min for the spheres to redistribute within the lesion and be pushed more distally by the blood inflow. After this waiting period, more embolic material was injected at the same flow rates. The total quantity of vials delivered at each session was recorded.

#### Patient Medication

The standard for a chemoembolization procedure with drug-eluting microspheres was administered [6, 7, 9]. Medication immediately before and during embolization included intravenous (IV) cefuroxim sodium/Zinacef 750 mg, IV metronidazole/Flagyl 500 mg, IV dexamethasone/Decadron 10 mg, IV ranitidine/Zantac 100 mg, IV ondacetron/Zofron 8–16 mg; and intramuscular pethidine 150 mg for the prevention of postembolization syndrome.

#### Patient Management

Hospital discharge was 24 h after embolization. Routine medication on discharge included the following: codeine/paracetamol 30/500 mg (Lonalgal) to be used only if pain occurred, ranitidine 100 mg p.o. daily, cefuroxime sodium 500 mg and metronidazol 500 mg p.o.q 8 h for 5 days after embolization. CEUS was routinely performed immediately before discharge in all patients for the detection of potential complications.

#### Doxorubicin Pharmacokinetics: Analysis in Plasma

Measurements of plasma levels of doxorubicin were made at baseline and at 5, 20, 40, 60, and 120 min, at 6, 24, and 48 h, and at 7 days after embolization to calculate maximum concentration levels of doxorubicin (Cmax) and area under the curve (AUC). Τhese measurements were also performed in three patients treated with c-TACE. The measurements were performed in patients with a single feeder because the time of the administration of the embolic suspension was shorter compared with those that required subsequent embolization of multiple feeders. Doxorubicin levels in plasma were evaluated by high-performance liquid chromatography (HPLC) coupled with mass spectrometry (MS). HPLC was performed using a Dionex Ultimate 300 0 system (Dionex, Germany) equipped with three pumps, a temperature-controlled column compartment, and an autosampler. A Waters Sunfire C8 column (3.5 μM, 2.1 × 50 mm) was used at a flow rate of 0.3 ml/min for sample retention. MS was performed on an API 4000 QTRAP LC-tandem MS/MS system fitted with a TurboIonSpray source and a hybrid triple quadrupole/linear ion trap mass spectrometer (Applied Biosystems, Concord, ON, Canada).

Plasma samples were stored at −80 °C until the day of the analysis. Samples were prepared for quantification by an established protocol of protein precipitation, collection of supernatants, evaporation, and resuspension into mobile phase before analysis [22]. Doxorubicin was quantified by LC-MS/MS analysis using doxorubicin standards (0.5, 1.0, 2.5, 5.0, 10.0, 25.0, 50.0, 100.0, 250, 500, 1,000, and 2,500 ng/ml) and on the addition of an internal standard for the generation of standard curves. The lower limit of quantification was 0.5 ng/ml using 0.1 ml of plasma.

### Data Analysis

Statistical significance was defined as a *p* value <0.05. Values for all continuous variables are quoted as mean, SD, minimum, and maximum throughout. Shapiro–Wilk test was used to evaluate the normality of distributions. Pharmacokinetics comparisons between the two groups (i.e., HepaSphere vs. c-TACE) were performed using Student *t* test (for maximum concentration) and Wilcoxon rank-sum test (for AUC). The AUC from baseline to 7 days was calculated using the linear trapezoidal method. Data processing and analysis were performed with SPSS 19.0 (Chicago, IL, USA).

## Results

The demographics, clinical characteristics, and tumour(s) are listed in Table 1. Median tumor diameter was 8 cm and mean was 8.3 ± 2.3 (range 4–14). BCLC stage was A for 7 patients, and the remaining 38 were BCLC stage B. Twenty-five patients were Child–Pugh class A (55.5 %), and 20 were Child–Pugh class B (44.5 %). No patients required additional medication or unscheduled visits. The mean number of vials received was 1.42 (range 0.5–2). In no case was additional embolic material given after completing the injection of HepaSphere 30–60 μm suspension because stasis was already achieved. The total number of chemoembolizations was 124. Segmental embolization was feasible in 86 sessions, and in 38 sessions, more than two segments were embolized. Mean hospitalization was 1.03 days (range 1–2).

**Table 1:** Demographics and tumor parameters of study participants

Age	69.7 ± 7.23 (range 56–81)
Sex (%)	
Male	34 (75.5)
Female	11 (24.5)
Etiology (%)	
HBV	33 (73.4)
HCV	4 (8.8)
HBV + HCV	6 (13.4)
Alchohol	1 (2.2)
Other (unknown)	1 (2.2)
Child–Pugh stage (%)	
A	25 (55.5)
B	20 (44.5)
BLC stage (%)	
BCLC A	7 (15.6)
BCLC B	38 (84.4)
ECOG score (%)	
0	25 (55.5)
1	20 (44.5)
Tumor location (%)	
Unilobar	37 (82.2)
Bilobar	8 (17.8)
No. of tumors (%)	
Single	21 (46.7)
<3	15 (33.3)
Multinodular	9 (20)
Tumor(s) diameter (cm)^a^	8.3 ± 2.3 (range 4–14)
Median (cm)	8.0
Tumor vascularity (%)	
Yes	41 (91.2)
No	2 (4.4)
Missing data	2 (4.4)

^a^Mean values

The rates of local response, including all dosage levels, are summarized graphically in Tables 2 and 3. Table 2 shows the rates of local response in the target lesions after each embolization session. Table 3 shows overall response and response of target and nontarget lesions as well as new lesions as required by mRECIST criteria. CR was achieved in 17.8 % of cases overall, reaching 22.2 % for the target lesion according to mRECIST criteria. Overall PR was seen in 51.1 %, SD in 20 %, and PD in 11.1 % of cases. Overall objective response (CR + PR) was seen in 68.9 % of cases. After a median follow-up of 15.6 months, the 1-year survival rate was 100 %.

**Table 2:** Rates of local overall response according to the mRECIST criteria at three follow-up visits 4 weeks after each embolization

Follow-up	CR	PR	SD	PD
First	0 (%)	24 (53.3 %)	21 (46.7 %)	0 (%)
Second	6 (%)	21 (13.3 %)	18 (46.7 %)	0 (40.0 %)
Third	8 (17.8 %)	23 (51.1 %)	9 (20.0 %)	5 (11.1 %)

**Table 3:** Rates of local response 1 month after the last chemoembolization session, including target and nontarget lesions and new lesions as required by mRECIST criteria

mRECIST	Overall response	Target lesion response	Nontarget lesion response	New lesions
	*n* (%)	*n* (%)	*n* = 40	*n* = 4
CR	8 (17.8)	10 (22.2)	5 (12.5)	
PR	23 (51.1)	21 (46.6)		
SD	9 (20)	10 (22.2)		
PD	5 (11.1)	4 (8.8)	4 (10)	
Non-PD			32 (80)	

### AFP Levels

**Fig. 1:**
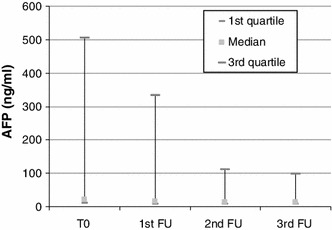
Blox plot of AFP levels at baseline and follow up visits 4 weeks after each embolization session

AFP levels during the study are shown in Fig. 1. Μean values before embolization (baseline) were 745.6 ± 2768.9 ng/ml. Mean values after embolization were 219.2 ± 377.6 ng/ml. Median values are seen in Fig. 1 in quartiles. There was a statistically significant decrease in levels of AFP after embolization, indicating the good response of the tumors to the embolization material (*p* < 0.0001).

#### Liver Enzymes

**Fig. 2:**
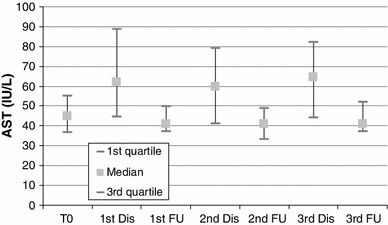
Blox plot of AST levels at baseline, discharge (24 h after each session) and at follow-up visits 3–4 weeks after embolization sessions

**Fig. 3:**
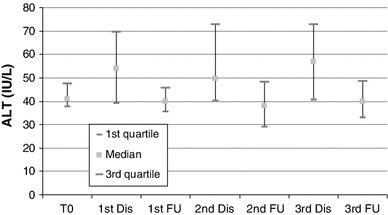
Blox plot of ALT levels at baseline, discharge (24 h after each session) and at follow-up visits 3–4 weeks after embolization sessions

**Fig. 4:**
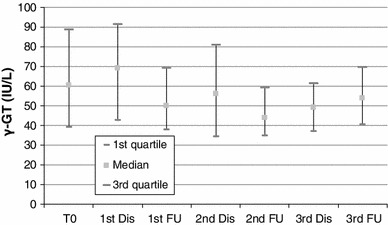
Blox plot of γ-GT levels at baseline, discharge (24 h after each session), and at follow-up visits 3–4 weeks after embolization sessions

**Fig. 5:**
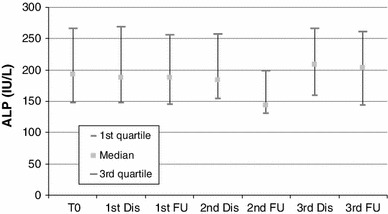
Blox plot of ALP levels at baseline, discharge (24 h after each session), and at follow-up visits 3–4 weeks after embolization sessions

There was an asymptomatic grade 1 increase in liver enzymes in 44–66 % of patients throughout the embolization sessions; AST and ALT baseline levels presented an increase immediately after each embolization session but not to a clinically significant level (Figs. 2, 3). At the visit 4 weeks after each embolization, the values of AST and ALT had returned to baseline levels. The rest of the liver enzymes presented no significant changes after embolization and at follow-up visits (Figs. 4, 5).

There was a 0 % 30-day mortality rate, and there were no grade 5 complications. The most common complication in this study was postembolization syndrome with an incidence of 18.54 %. No significant correlation was found between pain duration after embolization and the quantity of vials or the extent of embolization, with the exception of patients after their second embolization session. Those who received two vials of embolic material were more likely to have pain (*p* = 0.001). There was no statistically significant correlation between fever and the extent of embolization or quantity of vials. Other complications included a temporary increase in bilirubin in two patients (4.4 % grade 1 and grade 2), temporary ascites in one patient (2.2 %), grade 2 cholecystitis in one patient (2.2 %), and nontarget liver foci necrosis (grade 3) in one patient (2.2 %). There were no cases of liver abscess, alopecia, skin changes, and bone marrow suppression in this series. Endothelial damage, seen as stenosis or obstruction of subsegmental branches, was documented at repeat embolization in 4.4 % in our series [two patients (considered a grade 4 complication)].

**Fig. 6:**
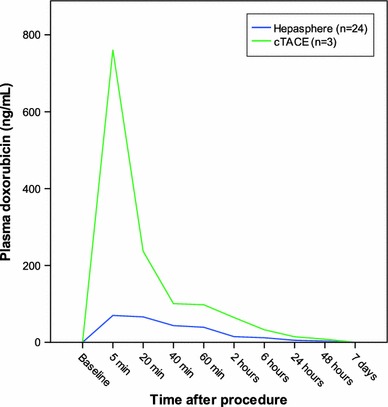
Plasma levels of doxorubicin measured immediately after embolization and at follow-up hours and days after the procedure. The* blue line* shows the levels after embolization with HepaSphere 30–60 μm loaded with doxorubicin at 50 mg/vial, and the* green line* shows the levels of doxorubicin at the same time spots after c-TACE with the same amount of doxorubicin. The *graph* indicates that there is no doxorubicin loss in plasma when embolizing with HepaSphere 30–60 μm, thus leading to less systemic toxicity

**Fig. 7:**
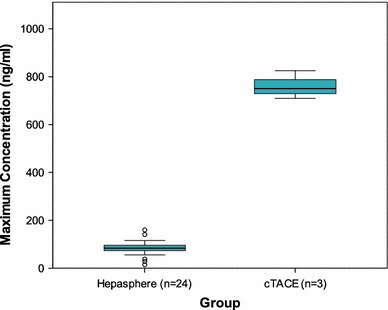
Cmax for HepaSphere and c-TACE showing the increased values of doxorubicin concentrations with c-TACE to a statistically significant level (*p* = 0.002). Cmax HepaSphere: 83.9 ± 32.1 ng/ml (mean ± SD). Cmax c-TACE: 761.3 ± 58.8 ng/ml (mean ± SD). Shapiro–Wilk test: HepaSphere = 0.451; c-TACE = 0.680) ≥ *p* = 0.002 (Student *t* test)

**Fig. 8:**
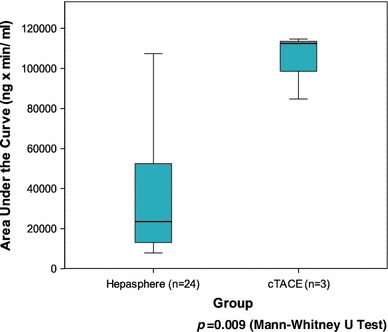
The AUC is displayed for HepaSphere 30–60 μm and c-TACE indicating the better pharmacokinetic profile of the former (*p* = 0.009 with Mann–Whitney U-test). The AUC was calculated using the linear trapezoidal method from baseline to 7 days. HepaSphere group (mean ± SD) 35,195 ± 27,873 (ng × min)/ml or 35.2 ± 27.9 (μg × min)/ml. c-TACE group (mean ± SD) 103,960 ± 16,652 or 103.9 ± 16.7 (μg × min)/ml

Pharmacokinetics were eventually performed in 24 patients who received HepaSphere 30–60 μm loaded with 50 mg of doxorubicin/vial and in 3 patients who received c-TACE with 50 mg of doxorubicin (pharmacokinetics were not performed at the lower escalation dose). Figure 6 depicts the doxorubicin levels over time in both the HepaSphere and c-TACE groups and clearly shows the lower levels of doxorubicin in plasma with HepaSphere 30–60 μm compared with c-TACE with the same amount of doxorubicin delivered, and therefore, the lower systemic toxicity of this type of chemoembolisation. The pharmacokinetic profile showed a peak doxorubicin concentration (Cmax) in plasma at 5 min (Fig. 6) after completion of the injection and was notably lower compared with the measurements of 3 patients who were embolized with c-TACE using the same total amount of doxorubicin (Cmax 83.9 ± 32.1 vs. 761.3 ± 58.8 ng/ml) (Fig. 7). Shapiro–Wilk test for HepaSphere was 0.451 and 0.680 for c-TACE (*p* = 0.002). It should be noted that samples of 3 patients were reanalyzed because doxorubicin levels were significantly greater than the suggested upper limit of quantification (250 ng/ml). The original values were confirmed with the use of an extended standard curve. Some of the samples were provided in duplicate. In that case, both of the duplicates were analyzed, and the mean values were used for the statistics and diagrams. The AUC was calculated using the linear trapezoidal method from baseline to 7 days (Fig. 8) and was 35,195 ± 27,873 and 103,960 ± 16,652 (ng × min)/ml for HepaSphere and c-TACE, respectively. Levels of doxorubicin in the plasma were consistently lower in the HepaSphere 30–60 μm group at all time points.

## Discussion

A number of studies with drug-eluting embolic materials have concluded that smaller calibers of microspheres are attractive because they achieve more distal embolization [6, 7, 12]. The study of Lee et al. [23] showed that only diameters <300 μm penetrate deep into the tumor microvasculature. Distal embolization is desirable to avoid hypoxia-induced neoangiogenesis [24, 25]. However very small diameters of drug eluting embolic agents, i.e., well below 100 μm, have not yet been tested clinically yet, whereas bland embolization with diameters, i.e., well below 100 μm, has been associated with increased rate of complications especially in large tumors [26, 27]. HepaSphere 30–60 μm is a newly developed small drug-eluting embolic agent. In a study in pigs, Dinca et al. [14] compared HepaSphere 30–60 μm with HepaSphere 50–100 μm and showed that the former achieves more distal embolization. Granulometry in this study showed that HepaSphere 30–60 μm expands ~4 times in saline with a mean diameter 197 ± 31 μm before loading with doxorubicin and 148 ± 45 μm after loading [14]. Deformation decreases after loading with a deformation factor of 7.9 ± 7.3 before and 5.6 ± 5.4 after loading [14]. In the same study it was shown that HepaSphere 30–60 μm showed successful loading with doxorubicin with a 38 times lower concentration of doxorubicin in the supernatant after loading compared with the initial preparation. In addition, it was shown that doxorubicin was released by the microsphere for a period of 1 month after embolization. They also showed that after embolization, the concentration of doxorubicin in tissue was high, whereas plasma levels were very low [14]. The two-step loading process of HepaSphere 30–60 μm, as suggested by the manufacturer, which was performed in this study, prevents fragmentation and aggregation of the microspheres as happened in the study of Jordan et al. [28]. The two-step loading process and the use of saline instead of water for injection allow the maximum binding of doxorubicin as has also been shown for the larger HepaSphere diameters [29].

The results of the present study shows that HepaSphere 30–60 μm is an effective embolic agent achieving major tumor necrosis, high rates of objective response (68.9 %), and a complete overall response 17.8 %, including all dosage levels of doxorubicin and varying lesion volumes. These results were obtained despite the fact that only 86 of 124 embolization sessions were segmental, whereas no segmental approach was feasible in the rest of the sessions. However, it must be pointed out that it is possible that the decreased re-embolization time may have augmented the results, and this must be taken into consideration when comparing local response with those of other studies.

The application of mRECIST criteria in this study gives the opportunity for further comparisons in the future because they are more detailed than European Association for the Study of the Liver (EASL) criteria and take into account both necrosis and size information. Our overall response results, with an objective response of 68.8 % and overall CR of 17.8 %, are quite encouraging and seem superior or comparable with the results of studies in which drug-eluting devices of 100–300 μm were used [6–8]. The size of HepaSphere 30–60 μm in vivo is 95–240 (148 ± 45) μm after loading with doxorubicin [14] and therefore is comparable with DC Bead 100–300 μm [14]. Despite the differences between mRECIST and EASL criteria (which was used in the previous studies), it is clear that CR and PD are absolutely comparable for assessing the value of the results of this study. Grosso et al. [30] using larger HepaSphere, achieved lower local response rates with 32 % objective response rates for lesions >5 cm and therefore comparable with our lesions—whereas in this study objective response was 68.9 %. The results of other studies with larger HepaSphere without doxorubicin show lower response rates [31, 32]. Seki et al. [33] using larger HepaSphere, achieved a CR of 12.6 and 43.7 % PR. Comparison with other studies with drug-eluting beads of similar diameter and treatment of similar tumor diameters in patients with mostly viral cirrhosis as in this study shows a better local response in our study [5–7]. From our results, it is noted that PD was not related to lack of response of the target tumor but rather to the development of new lesions. The fact that these results were achieved with a lower doxorubicin dosage (50 mg doxorubicin/vial) compared with the studies previously mentioned [5–7] may indicate the good embolic properties of HepaSphere 30–60 μm, including achievement of more distal embolization. The distal embolization was documented in the animal study of Dinca et al. [14] in which HepaSphere 30–60 μm provided a more distal occlusion and more dense distribution of microspheres in the embolized territory than HepaSphere 50–100 μm. Our hypothesis is that one contributing factor to the good local response may be the higher flexibility of HepaSphere 30–60 μm compared with other drug-eluting agents, which allows deeper penetration into the tumor microvasculature [22, 34–37].

Of paramount importance is the dilution of the embolic suspension after extracting the supernatant. In the first three patients in whom we used a denser suspension (loaded HepaSphere was added to only 10 ml of a mixture of contrast and saline), only a small quantity of the embolic material could feasibly be administered due to the aggregation of the spheres and proximal occlusion, whereas in the rest of the patients the higher dilution (total of 30 cc/vial) was used, thus allowing more embolic material to be delivered. The incremental injection used allowed the embolic agent to be carried further distally with blood flow. Furthermore, after waiting for a few minutes after initial stasis, it was possible to administer more sphere suspension, and it is speculated that during this waiting period, the spheres were pushed deeper into the tumor microvasculature by the blood flow (redistribution of spheres). Pharmacodilatation with nitroglycerin was used in this study before the injection of the embolic agent. It is only assumed that this contributed to more distal embolization; however, there is no definite documentation.

The pharmacokinetic profile in our study is quite similar to that of the studies of Varela et al. [3, 4] documenting clearly the principle of drug-eluting technology that leads to smaller systemic exposure of doxorubicin. The peak of doxorubicin levels in the patients embolized with HepaSphere was seen at 5 min. However, due to the slow injection rate of HepaSphere suspension, the peak of the maximum concentration may have occurred somewhat later than shown in Fig. 6 because there was a 20–25-min delay between the initiation of the administration of the embolic material and the acquisition of the blood sample (this is because the duration of the injection ranged between 6 and 15 min). Overall, levels of doxorubicin in plasma were lower compared with c-TACE at all time points (Fig. 6), a fact that documents lower systemic exposure to doxorubicin. This cannot be attributed to the smaller doses of doxorubicin because the total quantity was comparable in both types of embolization. A weakness, however, must be pointed out in that the c-TACE cases were very few, and no doxorubicinol measurements were taken.

Overall, the number of complications in this study is very small. There was no 30-day mortality and no grade 5 complications. These results show fewer complications compared with the 3 % periprocedural mortality reported by Grosso et al. [30] using HepaSphere 50–100 μm that in vivo has a larger caliber of 200–400 μm. Liver enzymes were increased for a few days after embolization and returned to normal in the following visit, a pattern that was also observed in other drug-eluting embolic studies [5–8, 31, 32] and animal data [35]. In this series, there was no abscess formation and a very low percentage of cholecystitis (2.2 %) compared with other drug-eluting microsphere studies [5–8]. This may be related to the qualities of the embolic material or to the learning process in using drug-eluting devices in the previous studies [5–8]. The incidence of reflux into the cystic artery in cases where the microcatheter was near was decreased by waiting a few minutes to perform the control angiography while the spheres moved more distally and redistributed. The use of vasodilatation before embolization may have added to the increased compliance of the vascular bed, thus preventing reflux through the Windkessel effect [34–37]. In no case was there a need for additional embolic material after the two vials of HepaSphere 30–60 μm, even in large tumors. This helped to prevent the overembolization that might have been responsible for abscess formation in studies of other drug-eluting embolic agents where additional bland embolic material was used [7, 9]. No toxicities were associated with systemic action of doxorubicin in this series, a fact that is well explained by the pharmacokinetic profile of HepaSphere 30–60 μm. In addition, the rate of postembolization syndrome was lower (18.54 %) than that reported in the literature of other drug-eluting microspheres [3–8]. This may be related to the nature of the embolic agent, the nontoxic effect on the adjacent healthy liver, or the analgesic treatment during and after the procedure.

Hepatic artery damage seen as stenosis or obstruction documented at repeat embolization was observed in only 4.4 % at the fourth- and fifth-order branches in our series, which is very low compared with c-TACE [38, 39]. Given the fact that the end point of embolization was stasis, this low rate of arterial damage indicates that the chemotherapeutic inside the sphere (because the supernatant was not used) does not affect the endothelium. Moreover, it shows that the loaded spheres move further forward from the incoming blood and are squeezed into a more distal position into the tumor, thus mitigating this occlusive effect. This was also acknowledged by the fact that waiting a few minutes after initial stasis showed that subsequently the stasis was cleared and that administration of more embolic material was feasible. Seki et al. [33] using larger HepaSphere, observed 9.1 % hepatic artery damage. Erinjeri et al. [40] in their retrospective study, reported 13 % arterial damage at the fourth- and fifth-order branches after four to five sessions of bland embolization with induced stasis with PVA or tris-acryl gelatin microspheres.

There are several limitations in this study: It is a single series prospective study with a small number of patients and does not offer comparison results with c-TACE or other drug-eluting material. In addition, in the pharmacokinetic study, only three patients were included for comparison of doxorubicin plasma levels after c-TACE as was performed in previous studies evaluating the pharmacokinetics of other drug-eluting embolic agents [3, 4].

In summary, HepaSphere 30–60 μm is a highly effective and safe embolic agent for intermediate-stage HCC with low systemic exposure to doxorubicin. Objective local response with an intended doxorubicin dosage of 50–100 mg reached 68.9 % without damage to adjacent healthy liver as evidenced by imaging and liver biochemistry. Further studies with a longer follow-up defining time to progression and survival should be planned.
